# Supplementary material for: An Advanced Protocol for the Quantification of Marine Sediment Viruses via Flow Cytometry
Source: Viruses. 2021 Jan 13;13(1):102. doi: 10.3390/v13010102 (PMC7828538; doi:10.3390/v13010102)
Supplement: Supplementary file 1 [file viruses-13-00102-s001.pdf]

Supplementary material

An advanced protocol for the quantification of marine sediment viruses via flow cytometry

Mara E. Heinrichs[1], Daniele De Corte[1], Bert Engelen[1], Donald Pan[2,3†*]

[1] Institute of Chemistry and Biology of the Marine Environment, Carl von Ossietzky University of Oldenburg, Germany

[2] Institute for Extra-cutting-edge Science and Technology Avant-garde Research (X-star), Japan Agency for Marine-Earth Science and Technology (JAMSTEC), Yokosuka, Japan [3] Department of Ecology and Environmental Studies, The Water School, Florida Gulf Coast University, Fort Myers, Florida, United States

[†] Present address

[*]Corresponding author: dpan@fgcu.edu

Supplementary File:

Protocol for the extraction of viruses from marine sediments and quantification via flow cytometry

For convenience, we provide this detailed protocol containing a hands-on manual for the extraction of viruses from marine sediments using Nycodenz and subsequent quantification via flow cytometry, respectively. Here, methods from several publications are combined and modified, namely the extraction method by Pan et al. [1], the DNase I treatment by Danovaro and Middelboe [2] and flow cytometric quantification according to Brussaard et al. [3]. Note that the protocol for flow cytometric quantification is referred to the application on the Accuri C6 flow cytometer (BD Biosciences) and might have to be adjusted. Prior to processing your samples, you may also adjust some settings to your unique sample set (e.g., extraction volume). For further information see the main text.

Supplementary References

1. Pan, D.; Morono, Y.; Inagaki, F.; Takai, K., An improved method for extracting viruses from sediment: detection of far more viruses in the subseafloor than previously reported. *Front Microbiol* **2019,** *10*, 878.
2. Danovaro, R.; Middelboe, M., *Manual of Aquatic Viral Ecology*; Wilhelm, S.W., Weinbauer, M.G., Suttle, C.A., Eds.; American Society of Limnology and Oceanography: Waco, TX, USA, 2010; Chapter 8; pp. 74–81.
3. Brussaard, C. P. D.; Payet, J. P.; Winter, C.; Weinbauer, M. G., *Manual of Aquatic Viral Ecology*; Wilhelm, S.W., Weinbauer, M.G., Suttle, C.A., Eds.; American Society of Limnology and Oceanography: Waco, TX, USA, 2010; Chapter 11; pp. 102-109.

Supplementary File

Protocol for the extraction of viruses from marine sediments and quantification via flow cytometry

I. Extraction of viruses from sediments

1. Preparation of chemicals

Prepare all solutions with MilliQ, filter through 0.02 µm syringe filters (Anotop, Whatman) and store in small batches (e.g., Falcon tubes) at 4°C.

- 30% and 50% Nycodenz solution (approximately 2 ml per sample)
- 2.5% NaCl solution (approximately 10 ml per sample)
- 100 mM pyrophosphate stock solution (approximately 1 ml per sample)

2. Preparation of material

Prepare following material per sample (the day) before extraction and store at 4°C until usage.

- 15 ml or 50 ml Falcon tubes: Add 4.7 ml of 2.5% NaCl and 300 µl pyrophosphate.
- Two 15 ml Falcon tubes with 1 ml of 50% Nycodenz at the bottom. Carefully pipette 1 ml of 30% Nycodenz on top of the 50% Nycodenz solution. Avoid mixing of the solutions so that two distinct layers are established.
- 1.5 ml sterile Eppendorf tubes: Add 0.5-0.9 ml of 2.5% NaCl, depending on the desired sample volume. The final volume should be 1 ml of slurry (see 3.1).

3. Extraction protocol

3.1 To create a slurry, mix the sediment sample with 2.5% of NaCl to a total volume of 1 ml. We recommend using 0.1-0.5 cm^3 of sediment, depending on the sample characteristics (e.g., organic carbon content and expected virus abundances).

3.2 Transfer the sediment sample to the NaCl / pyrophosphate solution and mix until homogenous.

3.3 Sonicate the sample mixture for one minute on ice.

3.4 Carefully layer the sonicated sample onto the Nycodenz gradient. Avoid disrupting the gradient layer. You may notice that denser particles already sink to deeper layers.

3.5 Centrifuge for 30 min at 2,900 x g and 4°C in a swinging bucket rotor to separate sediment particles and viruses.

3.6 Collect the liquid layers down to the 50% Nycodenz layer using a syringe and needle. Avoid collecting the 50% Nycodenz layer, as it contains the bulk sediment particles.

3.7 Filter the liquid through a 0.2 µm pore size PES syringe filter (or 0.45 µm filter, if you want to keep larger viruses) to remove remaining cells and particles.

3.8 For a second extraction round, add 300 µl of pyrophosphate to the Falcon tube that contains the slurry and 50% Nycodenz solution. Fill up the tube to 6 ml with 2.5% NaCl and repeat steps 3.3-3.7.

3.9 Pool the filtered extract. The volume of the combined extract should be approximately 12 ml.

3.10 If using epifluorescence microscopy to verify flow cytometric counts, split the virus extract; e.g., use three-quarters of the extract for flow cytometric quantification and one-quarter for epifluorescence microscopy.

3.11 For flow cytometry, gradually concentrate the virus extract by using Amicon centrifugal filters (100 k, Merck) according to the manufacturer's guidelines to a final volume of 0.5 ml. Wash the concentrate with NaCl.

3.12 Divide virus extract into 100-150 µl aliquots.

3.13 If using DNase I treatment, add DNase I (0.1-0.5 U/ml final concentration), incubate for 15 min in the dark at room temperature or 37°C. The enzymatic reaction will be stopped by the addition of gluataraldehyde.

3.14 Add glutaraldehyde (epifluorescence microscopy-grade) to a final concentration of 0.5%, incubate for 20-30 min at 4°C and flash freeze the sample using liquid nitrogen.

3.15 Analyze samples directly or store at -80°C until analysis.

II. Virus quantification via Flow Cytometry

4. Preparation of chemicals

Prepare chemicals in small batches. Autoclave chemicals in Schott bottles with membrane lids. Filter through 0.02 µm syringe filters (Anotop, Whatman) prior to use to ensure optimal purity.

TE buffer pH 8

- Used for reference blanks and dilution of samples
- Prepare TE buffer pH = 8 (e.g., TE stock solution, Sigma Aldrich)

MilliQ

- Used for rinsing and cleaning of the flow cytometer
- Filter autoclaved MilliQ with 0.2 µm syringe filter prior to use (Millex, Merck)

SYBR Green I

- Prepare 1:200 dilutions of the SYBR Green I manufacturer's stock solution (10,000 x concentrate) with autoclaved, 0.02 µm-filtered MilliQ
- Split the SYBR Green I working solution in 50 - 100 µl aliquots and freeze at -20°C
- To avoid fading, use a fresh aliquot and protect it from light during handling

5. Quantification via flow cytometry

5.1 Preheat heating block on 80°C.

5.2 Filter MQ through a 0.2 µm syringe filter (Millex, Merck) and TE buffer through a 0.02 µm syringe filter (Anotop Whatman) into Falcon tubes.

5.3 Prepare flow cytometer for measurement and rinse system with filtered MilliQ.

5.4 Set threshold for all samples by monitoring blanks and controls. Depending on the sensitivity of your flow cytometer, the threshold may vary around FL1-H: 550.

5.5 Gently thaw samples in an ice-bath. Keep samples on ice during analysis.

5.6 Dilute samples (at least 10-fold, better if greater than 30-fold) with TE buffer to a final sample volume of 435 µl. Aim for an event rate between 100 and 1000 events per s.

5.7 It is recommended to prepare your samples in batches of approximately six samples and one TE blank. Analyse your samples without interruption by measurement of other organisms or other dyes.

5.8 Add 5 µl of the SYBR Green I working solution to each sample at an interval of 3-5 min between each sample and mix by hand. Avoid vortexing, as this may result in virus decay.

5.9 Incubate stained sample at 80°C (heating block) for 10 min in the dark and subsequently cool the sample for 5 min at room temperature in the dark.

5.10 Measure sample for at least 2 min or collect at least 10,000 events at a flow rate targeting an event rate between 200 and 800 events per s.

5.11 Backflush once or twice after high abundance samples (> 1000 events per s) to avoid sample carryover. If necessary, rinse the system with a MilliQ washing step in between samples.

5.12 At the end of each measurement day, clean the flow cytometer according to the manufacturer's instructions.
